# Genetic Characterization of Archived Bunyaviruses and their Potential for Emergence in Australia 

**DOI:** 10.3201/eid2205.151566

**Published:** 2016-05

**Authors:** Bixing Huang, Cadhla Firth, Daniel Watterson, Richard Allcock, Agathe M.G. Colmant, Jody Hobson-Peters, Peter Kirkland, Glen Hewitson, Jamie McMahon, Sonja Hall-Mendelin, Andrew F. van den Hurk, David Warrilow

**Affiliations:** Author affiliations: Queensland Health Forensic and Scientific Services, Brisbane, Queensland, Australia (B. Huang, G. Hewitson, J. McMahon, S. Hall-Mendelin, A.F. van den Hurk, D. Warrilow);; Commonwealth Scientific and Industrial Research Organisation, Geelong, Victoria, Australia (C. Firth);; The University of Queensland, St. Lucia, Queensland, Australia (D. Watterson, A.M.G. Colmant, J. Hobson-Peters);; University of Western Australia, Nedlands, Western Australia, Australia (R. Allcock);; QEII Medical Centre, Nedlands (R. Allcock);; Elizabeth Macarthur Agriculture Institute, Menangle, New South Wales, Australia (P. Kirkland)

**Keywords:** bunyavirus, emergence, arbovirus, sequencing, phylogenetics, viruses, Australia

## Abstract

To better understand the diversity of bunyaviruses and their circulation in Australia, we sequenced 5 viruses (Gan Gan, Trubanaman, Kowanyama, Yacaaba, and Taggert) isolated and serologically identified 4 decades ago as members of the family *Bunyaviridae*. Gan Gan and Trubanaman viruses almost perfectly matched 2 recently isolated, purportedly novel viruses, Salt Ash and Murrumbidgee viruses, respectively. Kowanyama and Yacaaba viruses were identified as being related to members of a large clade containing pathogenic viruses. Taggert virus was confirmed as being a nairovirus; several viruses of this genus are pathogenic to humans. The genetic relationships and historical experimental infections in mice reveal the potential for these viruses to lead to disease emergence.

## Abstract 2

Genetic relationships between bunyaviruses from Australia and pathogenic bunyaviruses from elsewhere indicate emergence potential.

The family *Bunyaviridae* contains a diverse group of viruses; 100 species have been approved, and many others have yet to be classified (*1*). The family comprises 5 genera: *Orthobunyavirus*, *Phlebovirus*, *Nairovirus*, *Hantavirus*, and *Tospovirus* (*2*). Viruses of the first 4 genera are arthropod borne and cause infections of medical and veterinary importance; those of the last genus infect plants. Globally, these viruses are the etiologic agents of potentially fatal human infections such as Crimean-Congo hemorrhagic fever, Rift Valley fever, hantavirus pulmonary syndrome, severe fever with thrombocytopenia syndrome, and various sporadic viral encephalitides in the Americas (California encephalitis serogroup). Vectors of vertebrate-infecting bunyaviruses include mosquitoes, midges, sandflies, and ticks; rodents are involved in hantavirus transmission.

Human infections with bunyavirus are globally distributed. In Australia, serosurveys indicate that Gan Gan (GGV), Trubanaman (TRUV), and Kowanyama (KOWV) viruses can infect humans (*3*–*6*). Limited evidence indicates that bunyaviruses cause mild disease such as arthritis but that they do not present a serious threat to human health (*3*), thereby suggesting that species of bunyaviruses in Australia may be less virulent than those found elsewhere, despite their close relatedness to highly pathogenic species. An alternative hypothesis is that these viruses are poorly described and that the lack of knowledge and available diagnostic reagents are contributing to cases not being identified. Previous pathogenesis studies in murine models support this hypothesis. The symptoms of weanling mice infected with GGV, TRUV, and Yacaaba (YACV) viruses (all isolated during 1960–1980) indicate that these viruses are potentially neurovirulent (*7*). The recent discovery of a novel phlebovirus, genetically related to viruses from the Americas, that causes disease in humans illustrates the potential for emergence of viruses in that genus (*8*). Certainly, Australia harbors many other bunyaviruses for which genetic and serologic information is largely unknown (*7*). Hence, there is a need to address the dearth of knowledge of these viruses, to better understand their genetic relationships, and to facilitate the development of diagnostic reagents.

Two apparently novel mosquito-transmitted bunyaviruses, Salt Ash virus (SASHV) and Murrumbidgee virus (MURBV), were recently detected in mosquitoes collected in the Australian state of New South Wales (*9*). It was suggested that these viruses belong to the genus *Orthobunyavirus*, the first members of this genus to be sequenced outside the midge-transmitted Simbu serogroup that infect animals in Australia. In addition, a short sequence from a virus (designated Finch Creek virus) isolated from ticks collected from royal penguins on Macquarie Island, an Australian Antarctic Territory, indicated that the virus was probably a member of the genus *Nairovirus* (*10*).

Field studies are still in progress, but more work is needed to provide a better understanding of the diversity of bunyaviruses and their circulation. Unfortunately, obtaining funding for field studies can be difficult. A cost-effective alternative way to obtain material is to characterize archived material. Hence, for this study, we used archived isolates from field collections in combination with high-throughput sequencing technologies to expand our knowledge of bunyaviruses. This study revealed a genetic relationship between bunyaviruses from Australia and pathogenic bunyaviruses found elsewhere in the world, indicating emergence potential. More recent field isolates indicate that the described viruses are currently circulating, demonstrating the value of exploring archival collections.

## Methods

### Virus Collection, Isolation, and Culture

**Figure 1:**
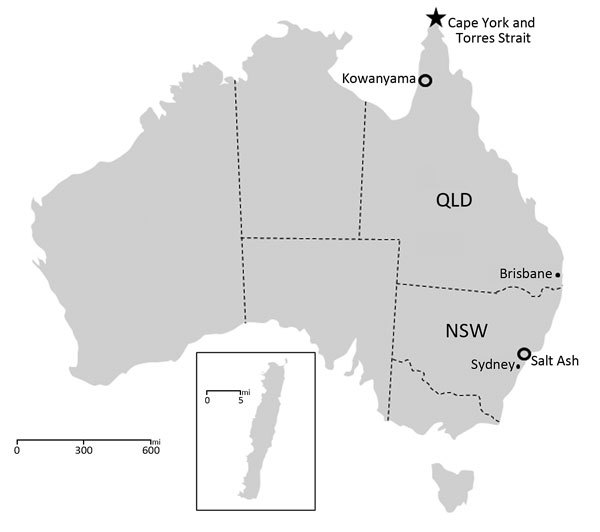
Bunyavirus collection and FTA card (Whatman, Maidstone, UK) sampling sites in Australia. Virus was collected from sites (open circles) in New South Wales (NSW), Queensland (QLD), and Macquarie Island (inset; 54°30S, 158°57E). Salt Ash is a town near Nelson Bay, NSW. Kowanyama is the site of the Mitchell River Mission, Queensland. FTA card sampling sites from Badu Island in the Torres Strait and Seisia and Bamaga on Cape York Peninsula are shown (star).

Most viruses in this study had been collected from mosquitoes and ticks of various species during 1953–1975 (Figure 1). Viruses were KOWV strain MRM1243, Taggert virus (TAGV) strain MI14850, TRUV thought to be strain MRM3630 (*11*,*12*), GGV strain NB6057, and YACV strain NB6028 (*13*). Viruses were originally isolated by use of intracerebral inoculation of mice or by culture on insect and mammalian cell lines (*13*,*14*); they were stored at −80°C until use in this study. After being thawed, isolates were grown on either C6/36 or Vero cells by using Opti-MEM (Thermo Fisher Scientific, Waltham, MA, USA) with 3% fetal bovine serum growth medium at 37°C (Vero) or 25°C (C6/36) under 5% CO_2_. GGV and TRUV isolates used for sequencing were from the Elizabeth Macarthur Agriculture Institute collection; all other archived material was obtained from the Queensland Institute of Medical Research collection. KOWV was reisolated from a homogenate derived from a pool of *Anopheles meraukensis* mosquitoes collected in 2001 as inoculum (*15*). The homogenate was passaged 1 time on C6/36 cells and then 1 time on Vero cells.

### Field Sampling 

We also collected mosquito expectorate samples on sugar-baited sample cards (FTA cards; Whatman, Maidstone, UK) by using methods previously described (*16*). Mosquitoes were from Badu Island in the Torres Strait and Seisia and Bamaga on the Cape York Peninsula (Figure 1). We extracted samples by using a QIAamp Viral RNA Extraction Kit (QIAGEN, Valencia, CA, USA) and screened them for viral RNA by using TaqMan reverse transcription PCR (RT-PCR) primers SAVSTF 5′-CAGTTTCTATCCTCTGGCTATTGGA-3′, SAVSTR 5′-GACGCAATGCCTTTTTTAGATATTG-3′, and probe SAVSTP 5′-FAM-ATTCAGAGCCAAACAAGACCCTGAGCAAG-TAMRA-3′.

### Archived Virus Testing

Virus was detected in samples by extraction of nucleic acids, followed by RT-PCR amplification with use of the following virus-specific primers: SAVSF1 5′-CATTGAAGTAAACCTACCAAGTGT-3′ and SAVSR1 5′-TCGAATATTGTATTATAATGATGT-3′, SAVMF1 5′-TTGTACAGTTGCTGGAAATTCAGT-3′ and SAVMR1 5′-TCTGGGTATGTTATACATATTTCT-3′, SAVLF1 5′-AGAAATAATCTAAAAAGAAGCTTA-3′and SAVLR1 5′-AAGTATAGGGTCCAATGCTGTCAA-3′ for SASHV small (S) (653 bp), medium (M) (765 bp), and large (L) (744 bp) segment amplification; and MVSF1 5′-CAGTGAGTTGAACCTAGGTAGCCT-3′ and MVSR1 5′-TCTTTTCTCTCTCCTCCTAATTTGAT-3′, MVMF1 5′-ATGCACACCTGCTTTAACTCAAAA-3′ and MVMR1 5′-GTAGGTGTGTTTATGCATATTTCA-3′, MVLF1 5′-AGGAACAATTTTAAACGTTCAATA-3′ and MVLR1 5′-TAAGATAGGATCACATGCAAATAA-3′ for MURBV S (652 bp), M (762 bp), and L (744 bp) segment amplification. We always included a no-template control.

### Viral Genome Sequencing

We sequenced the viral RNA genomes as described previously (*17*). In brief, we purified virus from tissue culture supernatant by using a combination of preferential nuclease digestion and ultracentrifugation, followed by sequence-independent amplification. A library was constructed from the products and sequenced on a Personal Genome Machine (Life Technologies, Carlsbad, CA, USA) by using 316 and 318 chips. 

### Electron Microscopy

We clarified the tissue culture medium containing cultured virus (at 3,000 × *g* for 10 min), layered the supernatant on a 20% sucrose cushion, and subjected it to centrifugation (100,000 × *g* for 16 h at 4°C). The supernatant was discarded, and the pellet was resuspended in sterile phosphate-buffered saline (20 μL). Virus-enriched resuspension was prepared for transmission electron microscopy on glow-discharged formvar-coated copper grids and negatively stained with 1% uranyl acetate. All images were obtained on a Tecnai F30 FEG-transmission electron microscope (FEI, Hillsboro, OR, USA) operating at 300 kV.

### Phylogenetic Analysis

We aligned the complete deduced amino acid sequences of each genome segment of KOWV, YACV, and TAGV with those of representative members of the *Orthobunyavirus* and *Nairovirus* genera available on GenBank. An additional nairovirus alignment was also created by using a short sequence fragment (<450 nt) of a highly conserved region of the L segment, which is the only region that has been sequenced for many nairoviruses. We generated alignments by using ClustalW implemented in Geneious version 8.1.6 (*18*), refined them manually, and removed ambiguously aligned regions by using the Gblocks program with default parameters (*19*). We constructed maximum-likelihood phylogenetic trees by using PhyML 3.0 (*20*), using the best-fit models of amino acid substitution as determined by the model selection procedures implemented through http://www.datamonkey.org (*21*). Phylogenetic relationships were determined by using a combination of nearest neighbor interchange and subtree pruning and regrafting branch swapping; 5 random starting trees were generated for each case. The phylogenetic robustness of each node was determined by using 1,000 bootstrap replicates and nearest neighbor interchange branch swapping.

## Results

### GGV and TRUV

Sequencing of the 5 virus isolates, previously determined by serologic testing to be members of the family *Bunyaviridae*, subsequently confirmed them to be bunyaviruses. The elucidated sequences of GGV and TRUV matched the sequences of 2 apparently novel mosquito-transmitted orthobunyaviruses identified in 2014 (*9*). These 2 viruses were GGV, which matched with SASHV, and TRUV, which matched with MURBV. Sequence alignment revealed 99% nt identity for fragments (all were >460 nt) of the S and M segments of GGV and SASHV and the S, M, and L segments of TRUV and MURBV (data not shown). This result suggests that these were in fact 2 viruses, not 4 viruses, and that the disparity resulted from different characterization methods used at the time of the separate isolations (serologic and sequence-based, respectively), which failed to identify an association. This finding is consistent with the fact that GGV was named after Gan Gan Army Base, which was located near the town of Salt Ash in New South Wales, where the virus was originally isolated from *Aedes vigilax* mosquitoes in 1970. TRUV was originally isolated from *Anopheles annulipes* mosquitoes at Mitchell River Mission in northern Queensland in 1965; Trubanaman was the original name of the Mission (*22*).

**Figure 2:**
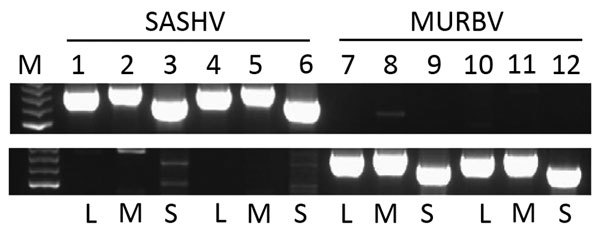
Sequences of Salt Ash (SASHV) and Murrumbidgee virus (MURBV) in archived stocks of Gan Gan (GGV) and Trubanaman viruses, respectively, from Australia. Archived material that was designated GGV (upper panel) and Trubanaman (lower panel) virus was extracted. This material was used in an assay designed to detect the small (S), medium (M), and large (L) segments of SASHV and MURBV viruses as indicated below the panels. Lane M, 100-bp ladder (Promega Corporation, Madison, WI, USA); lanes 1–3, GGV sample 1; lanes 4–6, GGV sample 2; lanes 7–9, MURBV sample 1; lanes 10–12, MURBV sample 2.

To verify that these 2 viruses had been renamed and were not simply mislabeled or incorrectly handled, we used the SASHV and MURBV GenBank genome sequences to design primer sets specific to each of the 2 viruses for detection by RT-PCR. Using assays to detect SASHV, we generated specific amplification products from material that had been designated as GGV in a separate archival collection held at the Queensland Institute of Medical Research (Figure 2) but not in material designated TRUV. Similarly, by using the MURBV assay, we generated products specific to that virus in samples designated TRUV from the Queensland Institute of Medical Research archival collection but not in material designated GGV. Hence, this evidence strongly suggests that these 2 viruses have been named twice, after independent isolations decades apart. It also indicates that both viruses have been circulating on the mainland of Australia for >40 years with little change. As part of a statewide surveillance program by the Public Health Virology Laboratory (Queensland Health Forensic and Scientific Services, Brisbane, Queensland, Australia), real-time RT-PCR detected GGV in mosquito expectorate that had been deposited onto nucleic acid sample cards (FTA cards) in a trap in the township of Seisia in northern Queensland. Hence, GGV is currently circulating in mosquito populations in that region. Unfortunately, we were not able to detect TRUV in this surveillance. However, the report by Coffey et al. (*9*) indicates that both viruses were recently circulating in New South Wales.

### KOWV and YACV

**Figure 3:**
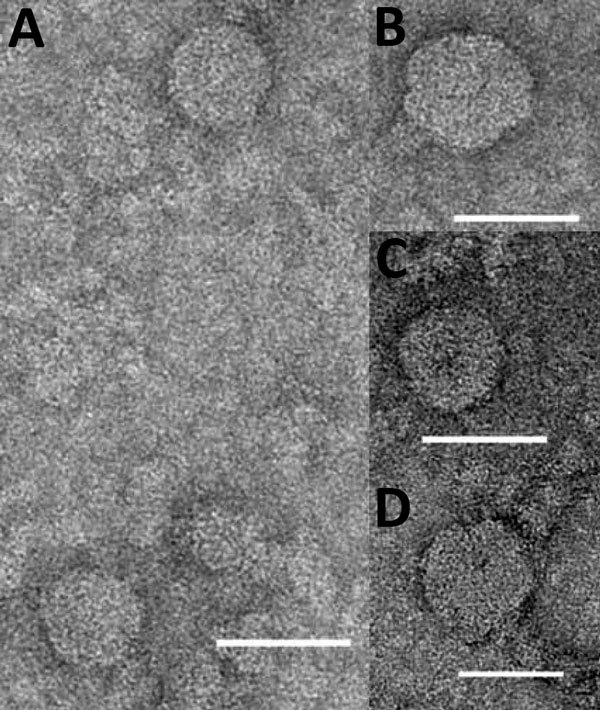
Negatively stained electron microscopic images of Kowanyama (A, B), Yacaaba (C), and Taggert virus (D) particles from Australia. Scale bars indicate 50 nm.

Two other viruses, KOWV and YACV, were also identified as being probable members of the genus *Orthobunyavirus*. These viruses were isolated from mosquitoes: *An. annulipes* (KOWV) in 1963 and *Ae. vigilax* (YACV) in 1970. Electron microscopy revealed multiple particles for both viruses, consistent with bunyavirus morphology (Figure 3). These particles were smaller than the 80–100 nm generally observed for bunyavirus particles; the small size was attributed to overnight sedimentation through a hyperosmotic sucrose cushion.

**Figure 4:**
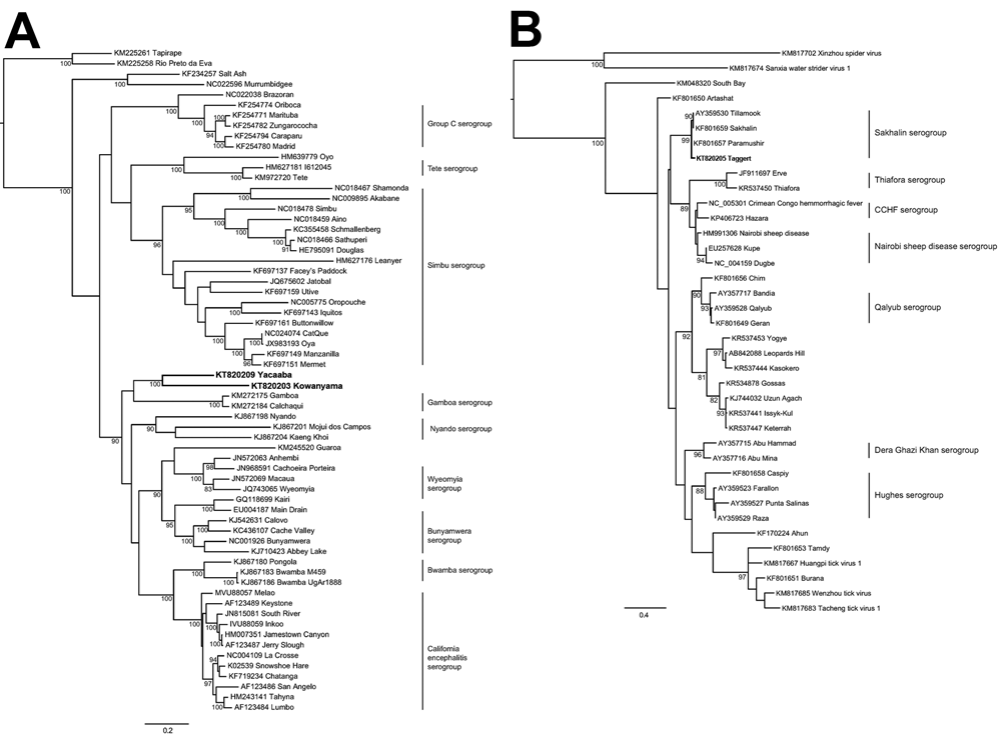
Phylogenetic trees including bunyaviruses from Australia. A) Relationship of Kowanyama and Yacaaba viruses (both in boldface) to other orthobunyaviruses constructed by using the predicted open reading frame sequence of the glycoprotein with a maximum-likelihood model. B) Relationship of Taggert virus (boldface) to other nairoviruses demonstrated by using the predicted open reading frame of a short fragment of the large segment (<450 nt) and a maximum-likelihood model. Virus serologic and genetic groups are shown to the right in each panel. Bootstrap values are shown as a percentage of 1,000 replicates. GenBank accession numbers are shown. CCHF, Crimean-Congo hemorrhagic fever. Scale bars indicate amino acid substitutions per site.

Phylogenetic analysis revealed that these 2 viruses form a clade that is most closely related to the Gamboa group of viruses (Figure 4, panel A; Technical Appendix Figures 1, 2). KOWV and YACV are also part of a larger clade, which includes the Nyando, Wyeomyia, Bunyamwera, Bwamba, and California encephalitis groups. Members of these groups have been associated with disease in humans and animals, which suggests the potential for disease emergence associated with these 2 viruses. In combination with historical neurovirulence studies in mice (*7*), this finding reveals the pathogenic potential of these recently characterized viruses. KOWV has been recently reisolated from a pool of mosquitoes (*An. meraukensis*) from 2001 (*23*), which indicates that KOWV was circulating in Queensland in that year (data not shown).

### TAGV

Phylogenetic analysis confirmed that TAGV is a nairovirus (Figure 4, panel B; Technical Appendix Figure 3). This virus was isolated from *Ixodes uriae* ticks collected on Macquarie Island in 1972. Nairovirus particles were observed by transmission electron microscopy (Figure 3). The assembled S segment RNA sequence also matched another more recent isolate from *I. uriae* ticks from Macquarie Island, designated Finch Creek virus (*10*). The authors of that work suggested that the virus may be related to TAGV but were unable to obtain a sample to confirm the relationship. A genetic relationship to other pathogenic members of the nairoviruses was also identified. The phylogenetic analysis of TAGV S, M, and L segments, in conjunction with its match to Finch Creek virus, confirms that it is a member of the genus *Nairovirus* and that the 2 viruses are closely related, if not strains of the same virus collected decades apart. TAGV provides another example of the problem of different characterization methods leading to duplicate designations for a single virus.

**Figure 5:**
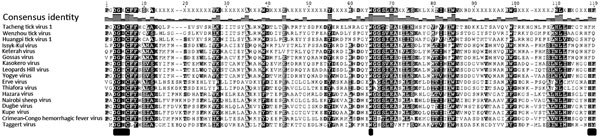
Taggert virus RNA-dependent RNA polymerase showing a viral homologue (vOTU) of the ovarian tumor domain. The alignment of nairoviruses shows a consensus sequence, which corresponds to the vOTU domain (*24*–*26*), which has been linked to virulence. The highly conserved residues, which include the catalytic triad residues, are indicated with a black box below each column.

Nairoviruses have a viral homologue (vOTU) of the ovarian tumor domain superfamily of proteases, which has been linked to virus virulence (*24*–*26*). vOTU has broad deubiquinating activity and is able to cleave the ubiquitin-like interferon-stimulated gene protein (ISG15) involved in host immune regulation, particularly the NF-κB (nuclear factor kappa light chain enhancer of activated B cells) signaling pathway (*24*–*26*). An alignment of the putative RNA-dependent RNA polymerase revealed that TAGV also has this domain (Figure 5), which includes 3 highly conserved blocks. Hence, TAGV may use vOTU activity to avoid innate host immune responses; this observation adds further support to the possibility that this virus may be pathogenic.

## Discussion

This study characterizes several bunyaviruses, originally collected decades ago, that are genetically similar to viruses known to be pathogenic. However, the drivers of infectious disease are not dependent solely on the genetics of the pathogen; they are also dependent on human and environmental factors, in which vector behavior plays a critical role. Previous infections of weanling mice established the neurovirulence potential of GGV, TRUV, and YACV (*7*). With regard to transmission, GGV and YACV were isolated from *Ae. vigilax* mosquitoes, which are widely distributed in coastal regions and are one of the primary vectors for Ross River virus transmission in Australia (*27*). Future transmission studies are needed to determine the vector competence of mosquito species to transmit GGV and YACV to humans, enabling an objective assessment of their threat to human health. The mosquito *An. annulipes*, from which TRUV and KOWV were isolated, only rarely feeds on humans (*28*) but may play a role in enzootic transmission. Furthermore, laboratory-based vector studies revealed that TRUV and KOWV replicated in *Ae. vigilax* and *Culex annulirostris* mosquitoes, respectively (*29*).

Intracerebral inoculation of TAGV into “infant” mice caused death, suggesting some level of virulence (*30*). In addition, a vOTU domain, which is a conserved region in the RNA polymerase of nairoviruses, was demonstrated to be present in TAGV. This observation and the genetic relationship between TAGV and other known pathogenic nairoviruses, suggest that TAGV may also be virulent. With regard to transmission, the *I. uriae* tick vector for TAGV has a circumpolar distribution on both hemispheres (*31*) and has been observed to occasionally bite humans (*11*). However, a limited serosurvey of Macquarie Island staff in the same study detected no antibodies to the virus in any person. The virus is yet to be detected on the Australia mainland, but it is potentially a risk to persons exposed to seabirds, the natural host of *I. uriae* ticks, which live at or have migrated through higher latitudes.

Surveillance and characterization of arboviruses are needed to better elucidate virus ecology, understand how viruses evolve, and be better prepared diagnostically if they ultimately emerge. The use of high-throughput sequencing technologies will mean that sequences for the design of diagnostic reagents to determine the virulence of these viruses can now be used. The use of archived material in this sequencing study shows the previously identified genetic stability of the arboviruses (*32*). When contemporaneous material was available, strains were found to vary by a small percentage only, for multiple genome segments, over >4 decades. This genetic stability is remarkable for RNA viruses and has previously been recognized for other arboviruses such as alphaviruses and flaviviruses. It has been attributed to the selection pressure exerted by the insect and mammalian hosts in the complete virus life cycle (*32*). It also demonstrates the value of collecting, storing, and analyzing archived material, which can reveal unexpected relationships when new technologies become available.

This work highlights an issue arising from the recent use of new high-throughput sequencing technologies, that is, the designation of recently collected virus isolates as novel material and their renaming. This issue arises from the fact that material collected before the 1980s was identified by use of serologic methods, whereas characterization of more recent isolates is primarily based on sequence analysis. To further illustrate this point, since this work was completed, another orthobunyavirus isolate from the Northern Territory in Australia, Buffalo Creek virus, was reported as being the same species as MURBV in the Mapputta group (*33*). Hence, TRUV, MURBV, and Buffalo Creek virus should all be considered the same virus. Although our study identifies a few regional examples, it is expected that this phenomenon is probably global. Workers should be aware of this possibility and should take steps to minimize the issue by sequencing archived material as well as using serologic techniques when available.

The risk for emergence of viruses, such as the ones described in this article, increases as population and growth pressures lead to development of previously undisturbed regions and concomitant exposure to native biota. This issue is especially of concern in Australia, where there is a drive to expand into the tropical northern part of the country. An additional consideration is that some persons may be exposed to insect vectors through research, mining, and other occupational activities, thereby increasing their risk for infection. In such instances, one might expect sporadic disease. The degree to which cases of sporadic disease represent a risk for spread to the community will depend on the factors affecting virulence but also on the degree of symptomatic disease and the rate at which these agents adapt to new mammalian hosts—only time will tell. But forewarned is forearmed, and surveillance remains the best preparation for future threats.

Technical Appendix Phylogenetic trees of orthobunyaviruses and nairoviruses.
