# Supplementary material for: Genetic Characterization of Archived Bunyaviruses and their Potential for Emergence in Australia
Source: Emerg Infect Dis. 2016 May;22(5):833–40. doi: 10.3201/eid2205.151566 (PMC4861517; doi:10.3201/eid2205.151566)
Supplement: Technical Appendix — Phylogenetic trees of orthobunyaviruses and nairoviruses. [file 15-1566-Techapp-s1.pdf]

Article DOI: http://dx.doi.org/10.3201/eid2205.151566

Genetic Characterization of Archived Bunyaviruses and their Potential for Emergence in Australia

Technical Appendix

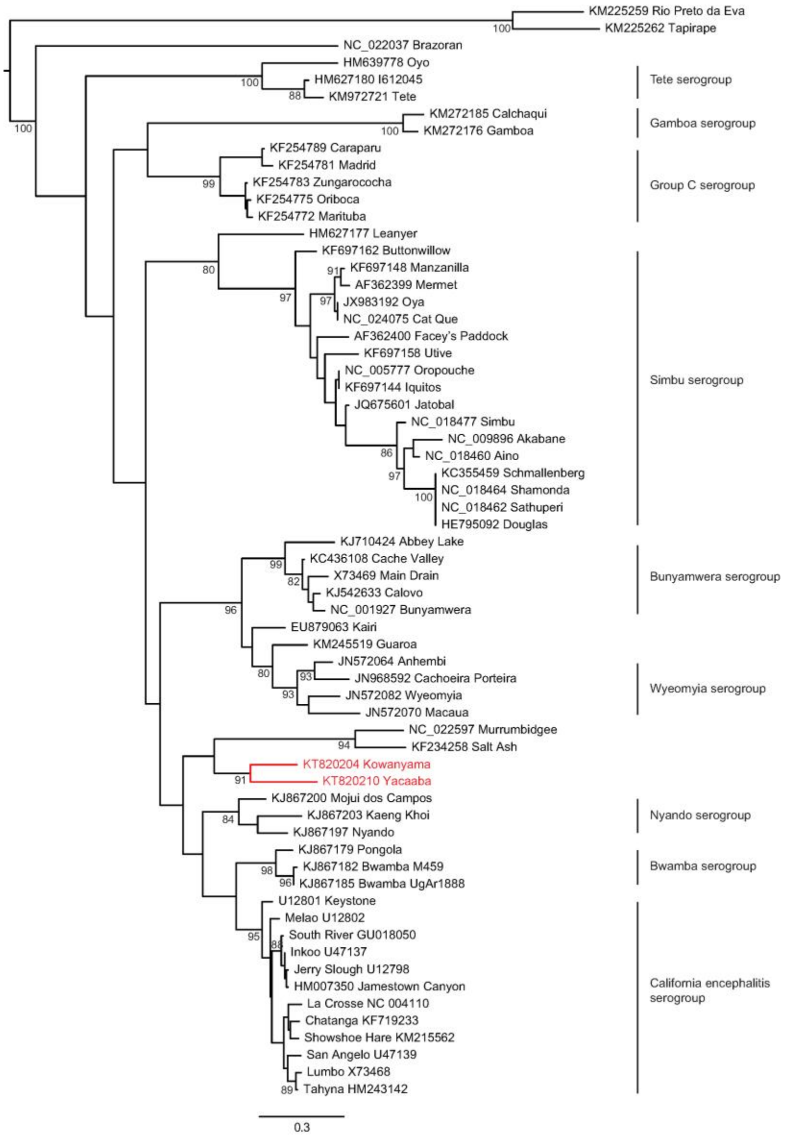

Technical Appendix Figure 1. Phylogenetic tree of the orthobunyaviruses based on the S segment ORF. The relationship of KOWV and YACV to other orthobunyaviruses using the predicted ORF sequence with a maximum likelihood model. GenBank accession numbers are shown.

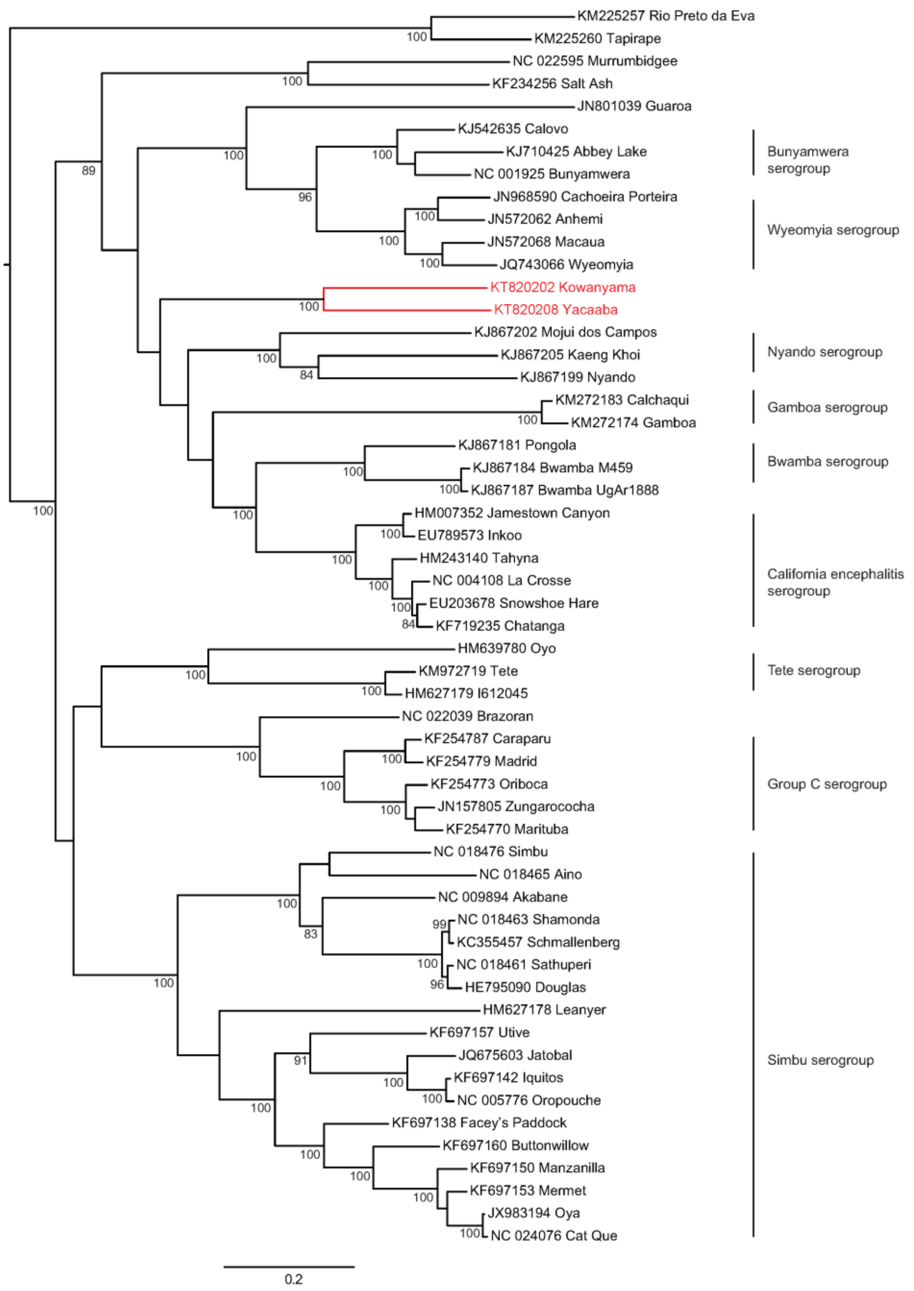

Technical Appendix Figure 2. Phylogenetic tree of the orthobunyaviruses based on the L segment ORF. The relationship of KOWV and YACV to other orthobunyaviruses using the predicted ORF sequence with a maximum likelihood model. GenBank accession numbers are shown.

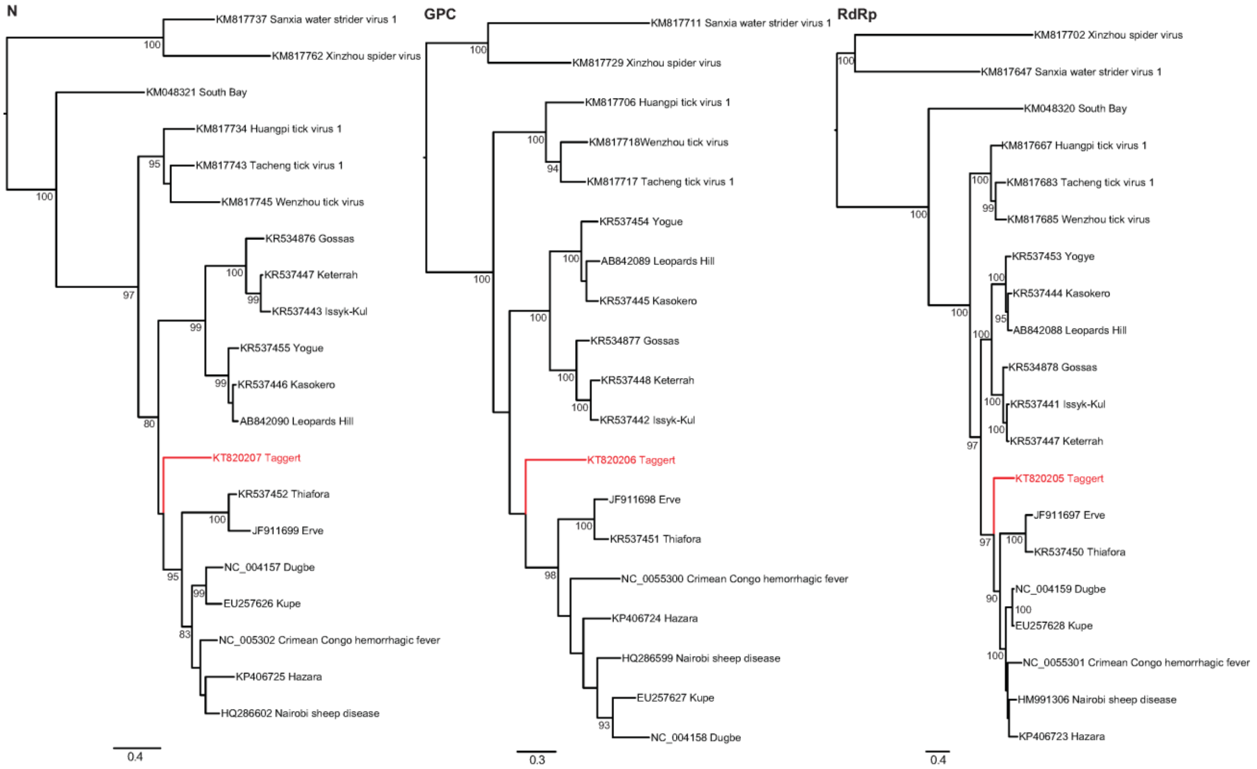

Technical Appendix Figure 3. Phylogenetic trees of the nairoviruses based on the complete S, M and L ORFs. The relationship of TAGV to other orthobunyaviruses using the predicted ORF sequence with a maximum likelihood model. GenBank accession numbers are shown.
